# Multisensory Integration Strategy for Modality-Specific Loss of Inhibition Control in Older Adults

**DOI:** 10.3390/ijerph15040718

**Published:** 2018-04-11

**Authors:** Ahreum Lee, Hokyoung Ryu, Jae-Kwan Kim, Eunju Jeong

**Affiliations:** 1 Department of Industrial Engineering, Hanyang University, 222 Wangsimni-ro, Seongdong-gu, Seoul 04763, Korea; ahrmlee@hanyang.ac.kr; 2 Department of Arts and Technology, Hanyang University, 222 Wangsimni-ro, Seongdong-gu, Seoul 04763, Korea; hryu@hanyang.ac.kr; 3 Smart Factory Business Division, Samsung SDS, 35 Olympic Ro, Seoul 05510, Korea; jaekey.kim@samsung.com

**Keywords:** inhibitory control, multisensory integration, audio-visual contour identification, ageing, modality-specific effect

## Abstract

Older adults are known to have lesser cognitive control capability and greater susceptibility to distraction than young adults. Previous studies have reported age-related problems in selective attention and inhibitory control, yielding mixed results depending on modality and context in which stimuli and tasks were presented. The purpose of the study was to empirically demonstrate a modality-specific loss of inhibitory control in processing audio-visual information with ageing. A group of 30 young adults (mean age = 25.23, Standard Deviation (SD) = 1.86) and 22 older adults (mean age = 55.91, SD = 4.92) performed the audio-visual contour identification task (AV-CIT). We compared performance of visual/auditory identification (Uni-V, Uni-A) with that of visual/auditory identification in the presence of distraction in counterpart modality (Multi-V, Multi-A). The findings showed a modality-specific effect on inhibitory control. Uni-V performance was significantly better than Multi-V, indicating that auditory distraction significantly hampered visual target identification. However, Multi-A performance was significantly enhanced compared to Uni-A, indicating that auditory target performance was significantly enhanced by visual distraction. Additional analysis showed an age-specific effect on enhancement between Uni-A and Multi-A depending on the level of visual inhibition. Together, our findings indicated that the loss of visual inhibitory control was beneficial for the auditory target identification presented in a multimodal context in older adults. A likely multisensory information processing strategy in the older adults was further discussed in relation to aged cognition.

## 1. Introduction

Ageing is a process that leads to a weakening of perceptual and cognitive capacity. With a decrease in the accuracy of sensory perception, older adults show declines in various type of cognitive functions (e.g., attention, working memory, and executive control) [1], which further have significant effects on everyday life [2,3,4,5,6]. A reduced ability to inhibit irrelevant information, for example, is considered as a form of cognitive decline with ageing [7,8]. The elderly are less capable of adequately filtering irrelevant sensory noises (i.e., distractions) and are hence more susceptible to distraction than younger adults [9].

Such age-related changes need to be considered in relation to multisensory processing since our experience is multimodal in nature. Multisensory integration refers to a process by which inputs from two or more senses are combined to form a product that is distinct from, and thus cannot be easily broken down to, each component [10]. At an earlier stage, two or more sensory inputs are individually processed [11,12,13] and later higher-order cognition involves a cross-modal attention transfer [14,15,16]. Multisensory integration orchestrates multiple sources of inputs, so a well-known phenomenon, the McGurk effect [17], represents elderly participants’ possible communication problems with multisensory audio-visual stimuli [18].

Our brain is highly plastic and reversible, however, and develops a compensatory strategy when necessary [19]. Older adults show prominent neurological deactivations in the primary sensory cortices [3,5,20,21,22,23,24], and instead show simultaneous activation of additional brain networks, such as the prefrontal and parietal cortices [2,6,25]. A recent study reported that performance on a word memory task among older adults was enhanced and yielded better results as compared to younger adults when distracting words were relevant to target words [26,27]. That is, the older adults are likely to process multiple sources of information in a more ‘fanned out’ rather than ‘zoomed in’ manner, thereby allowing them to utilize relevant information from distractions in order to perform a target task. This tendency can be interpreted as an effective compensatory strategy that balances the loss of attention control (or inhibitory control) with ageing, which is a central theme that the present study aimed to demonstrate empirically. 

Indeed, research on multisensory integration with ageing has been made in the audio-visual paradigm (i.e., one modality as target and the other as a distracting non-target) using static visual and auditory stimuli. A series of studies examined the modality-specific effect in audio-visual information processing in aged cognition. They found that visual target detection against the auditory non-target was intact, but filtering visual distraction against auditory target was hampered [28,29,30]. In a similar vein, Guerreiro and Van Gerven [31] reported that more distraction occurred for the elderly than the young adults by visual irrelevant stimuli during the auditory n-back task. In a subsequent study, Guerreiro et al. [32] again established the asymmetric effect of ageing for cross-modal distractibility. That is, visual interference is greater in the elderly during the auditory target detection, while auditory interference does not significantly affect visual attention performance. In effect, it seems that the suppression processing in the auditory modality occurred at an earlier stage [33,34], as evidenced by P50 components [34,35], and ageing does not make this poorer. 

Recent evidence also suggests that older adults exhibit impaired suppression of visual distraction at the cortical level [29,36]. Since cognition in the elderly is associated with reduced ability at the cortical level, this is likely to be one of the main causes of failure in inhibiting visual distraction. Meanwhile, older adults showed relatively intact suppression of the auditory distraction due to the early filtering mechanism [32]. That is, information from auditory distraction is less likely to be taxed when unattended, while visual distraction shows a greater chance of being affected at the cortical-level perception. This is in accordance with the findings showing increased susceptibility of older adults to visual distraction, even when it is unattended. As such, asymmetric sensitivity to the unattended stimuli can play an important role in multisensory integration information processing [31].

Another series of studies, conversely, reported a salient effect of auditory interference with ageing. Lutfi-Proctor et al. re-designed the conventional Stroop test for an audio-visual version and found that auditory interference (i.e., semantics or phonetics distraction) heavily deteriorated the visual task performance [37]. Interestingly, Leiva et al. [38] examined the saliency of auditory interference in the different age groups, demonstrating that the elderly seemed to show an audio-sensitivity effect in the cross-modal task but not in the unimodal task. More recently, Stothart and Kazanina [39] conducted an event-related potential (ERP) study to examine peripheral and cortical involvement of auditory interference between different age groups. Their findings showed that increased vulnerability to auditory distraction was due to the cortical inability to inhibit peripheral processing. The combination of increased early sensory responses P50 (i.e., a positive evoked potential occurring approximately 50 ms after the stimulus presentation) and N1 (i.e., a negative evoked potential occurring approximately 100 ms after the stimulus presentation) , absent or strongly reduced N2 to standard stimuli, and an increased P3a to deviant stimuli in the elderly participants indicated a decreased ability to inhibit responses to regular repeating information, and greater attentional capture from rare task-irrelevant information. All these findings implied that older adults are more likely to weigh auditory information even when it was unattended, which contradicts Guerreiro and her colleagues’ findings [7]. 

The purpose of the study was, thus, to empirically demonstrate the modality-specific effect of audio-visual information processing with a selective attention and an inhibitory control framework with ageing. For this, we noted that both visual and auditory information can form perceptual contours, and developed a nonverbal, audio-visual contour identification task (AV-CIT). Using the AV-CIT, we also aimed to determine whether the characteristics of aged cognition (i.e., the loss of inhibitory control) would play a modality-specific role in processing audio-visual information. 

## 2. Materials and Methods 

### 2.1. Participants

Thirty younger adults and thirty older adults participated in this study. The younger adults were voluntarily recruited from the course titled “Introduction to Psychology” at Hanyang University, Seoul, South Korea. The older adults, aged 40–60 years, were recruited through online advertisements. Eight older adults were excluded because they did not complete the whole experimental session. Thus, the data from 30 younger adults (Mean (M) = 25.23 years, Standard Deviation (SD) = 1.86, 15 women) and 22 older adults (M = 55.91 years, SD = 4.92, 7 women) were used in this analysis. All participants had no medical history of sensory defects and reported normal vision and hearing capacities. The younger group had significantly fewer years of formal education (M = 14.53 years, SD = 1.65) than the older adult group (M = 15.63 years, SD = 1.18) (t(50) = −2.67, *p* < 0.05). All participants were given instructions regarding the experiment and a consent form prior to the study. This study was approved by the Clinical Research Ethics Committee of Hanyang University Hospital (HYUH 2013-08-017-002).

### 2.2. Stimulus Characteristics 

#### 2.2.1. Auditory Stimuli

**Figure 1:**
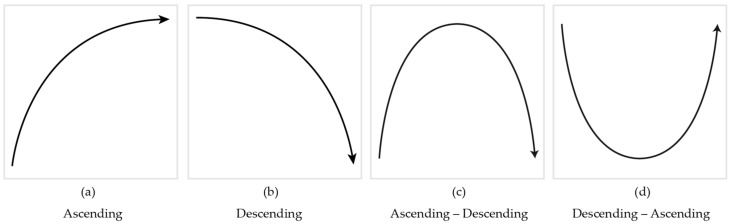
The contours of auditory stimuli.

All sounds were generated by a musical instrument digital interface (MIDI) synthesizer (YAMAHA DGX 230, Japan) with a digital audio workstation and MIDI sequencer (Logic Pro X, Apple Inc., Cupertino, CA, USA). The auditory stimuli were four different types of melodic contours (see Figure 1), adopted from a previous study [40] as follows: (1) a series of tones with 261.6 Hz, 392.0 Hz, 523.3 Hz, 587.3 Hz, 659.3 Hz, 784.0 Hz, 1046.5 Hz, 1174.7 Hz, and 1318.5 Hz for an ascending contour; (2) 1318.5 Hz, 1174.7 Hz, 1046.5 Hz, 784.0 Hz, 659.3 Hz, 587.3 Hz, 523.3 Hz, 392.0 Hz, and 261.6 Hz for a descending contour; (3) 261.6 Hz, 392.0 Hz, 523.3 Hz, 587.3 Hz, 659.3 Hz, 784.0 Hz, 1046.5 Hz, 1174.7 Hz, 1318.5 Hz (peak point), 1174.7 Hz, 1046.5 Hz, 784.0 Hz, 659.3 Hz, 587.3 Hz, 523.3 Hz, 392.0 Hz, and 261.6 Hz for ascending to descending contour; and (4) 1318.5 Hz, 1174.7 Hz, 1046.5 Hz, 784.0 Hz, 659.3 Hz, 587.3 Hz, 523.3 Hz, 392.0 Hz, 261.6 Hz (bottom point), 392.0 Hz, 523.3 Hz, 587.3 Hz, 659.3 Hz, 784.0 Hz, 1046.5 Hz, 1174.7 Hz, and 1318.5 Hz for descending to ascending contour, in conjunction with the six different time periods (500/1000/1500/2000/2500/3000 ms). The four different melodic contours were randomly presented during the experiments. 

#### 2.2.2. Visual Stimuli

**Figure 2:**
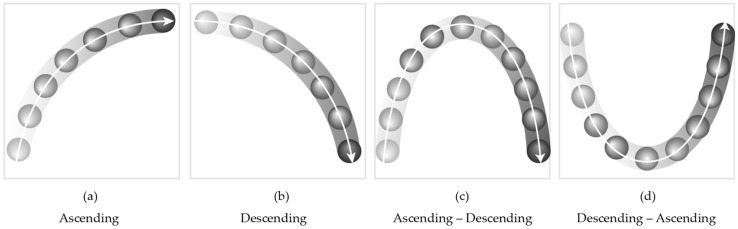
The contours of visual stimuli.

The visual stimuli with dynamic balls were designed to be equivalent to the melodic contour (see Figure 2): (1) ascending, (2) descending, (3) ascending to descending, and (4) descending to ascending, along with the six different time periods (500/1000/1500/2000/2500/3000 ms). 

### 2.3. Audio-Visual Contour Identification Task 

Our experimental task, AV-CIT, consisted of four successive contour identification subtasks: (1) Unimodal Auditory Contour Identification Task (Uni-A), (2) Unimodal Visual Contour Identification Task (Uni-V), (3) Multimodal Auditory Contour Identification Task with Visual Distraction (Multi-A), and (4) Multimodal Visual Contour Identification Task with Auditory Distraction (Multi-V). Each CIT was performed 24 times, with a two-minute break between the four CITs. 

The four types of contour directions (see Figure 1 and Figure 2) were randomly played with various time periods (i.e., 500, 1000, 1500, 2000, 2500, and 3000 ms). This was done to confirm if the presentation time of the stimuli is seemingly associated with both ageing and modalities. In both Uni-A and Uni-V conditions, the participants were asked to identify the contour direction of either the auditory stimulus they heard, or the visual stimulus they saw, by clicking the button on the tablet as quickly as possible. For these two subtasks, no distracting stimulus was provided and the target modality was instructed prior to the test. In the Multi-A and Multi-V conditions, the participants were asked to selectively attend on either the auditory target contour (Multi-A) or the visual target contour (Multi-V). For the Multi-A, the visual stimulus was given as a distracting non-target; the Multi-V employed the auditory stimulus as a distracting non-target. Unlike both Uni-A and Uni-V conditions, Multi-A and Multi-V involved both target and non-target stimuli, so the instruction of the target modality was not too obtrusive. For this purpose, the target modality was given by the background color (a black background indicated an auditory target and a visual non-target, and a white background indicated a visual target and an auditory non-target). 

### 2.4. Procedure

**Figure 3:**
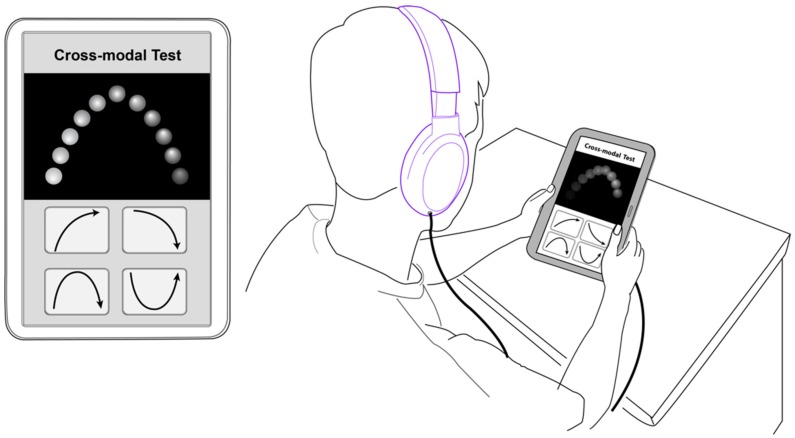
Audio-visual contour identification task and the experimental setting.

The experiment was performed in a sound-proof and light-controlled room to minimize other distractions and ensure that full attention was committed to the test. The participants were individually tested. They were asked to hold the tablet with their non-dominant hand and conduct the experiment with their dominant hand. All the participants put on headphones. The experimental setting is shown in Figure 3. Prior to the main experiment, instructions were given and the participants performed two or three trials with the same experimental apparatus. In the main experiment, the participants individually performed the tasks without the presence of the experimenter. They were asked to choose the correct answer on the tablet as quickly as possible. The entire experiment took around 20 min. Participants performed the experimental task on a 10.1-inch tablet personal computer with a resolution of 1280 × 800 pixels. The server pages (i.e., algorithm, data connection, and visualization) were developed by PHP, and MySQL was also used for storing data such as demographic information, test questions, reaction times, and answers. 

### 2.5. Data Analysis

For the data analysis, the response time and the accuracy of each test were collected. The response times (milliseconds) and accuracy (percentage of correct answers) were analyzed by two-way within-subject analysis of variance (ANOVA). Missing values as a result of technical problems were handled by using the linearly predicated value [41]. As the sphericity was not met, Greenhouse-Geisser corrections [42] to the degrees of freedom were applied instead. The original degrees of freedom are presented with the corrected p and ε values in the Results. When examining the pairwise contrasts, the Bonferroni approach [43] was adopted. All statistical analyses were performed with SPSS version 21.0 (SPSS Inc, Chicago, IL, USA). 

## 3. Results

### 3.1. Comparing the Performance of the Four Audio-Visual Contour Identification Tasks 

**Figure 4:**
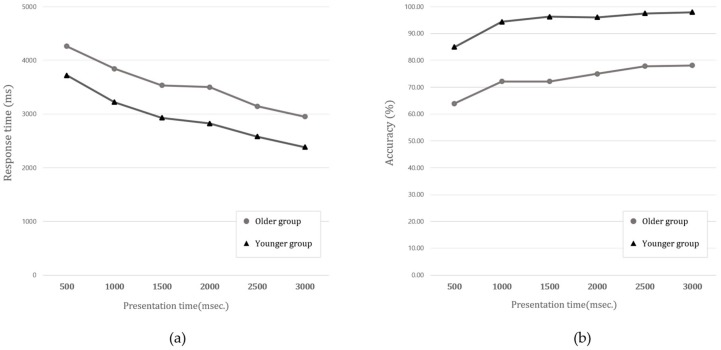
Changes in (**a**) response time and (**b**) accuracy across presentation time.

Figure 4 shows the performance of the two age groups on all the four CITs. As the presentation time increased from 500 to 3000 ms, the response time on all the four CITs tended to consistently decrease. The accuracy values also showed a similar pattern to the response time, and they increased with the presentation time. Descriptive analyses indicated that both the response time and accuracy had a consistent linear effect along with the test presentation time (i.e., decreased response time (RT) and increased accuracy with a longer presentation time) (see Table 1). This tendency was similar for the both age groups (see Figure 4). For these reasons, we aimed to ignore the effect of time and aggregate all the data across the presentation time. For further analysis, we generated a new data set into Group (younger vs. older), Target modality (auditory vs. visual), and Task type (uni vs. multi). 

**Figure 5:**
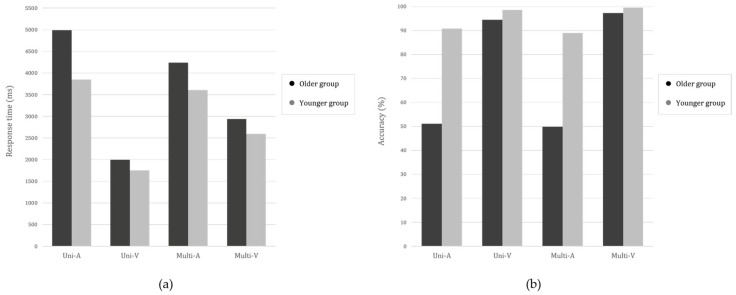
Changes in (**a**) response time and (**b**) accuracy across audio-visual contour identification tasks (AV-CITs). Uni-A: Unimodal Auditory Contour Identification; Uni-V: Unimodal Visual Contour Identification Task; Multi-A: Multimodal Auditory Contour Identification Task with Visual Distraction; Multi-V: Multimodal Visual Contour Identification Task with Auditory Distraction.

Next, we performed a three-way mixed ANOVA using the target modality (auditory vs. visual), task type (uni vs. multi), and Group (younger vs. older) (Table 2). As depicted in Figure 5a, with regard to response time, there was a significant main effect of group (F_(1,50)_ = 11.96, *p* < 0.05, η_p_^2^ = 0.19), indicating that younger adults performed better than the older age group in all tasks (*p* < 0.01). The main effect of target modality was significant (F_(1,50)_ = 229.61, *p* < 0.001, η_p_^2^ = 0.82), indicating that both groups required significantly shorter time in the Uni-V than in the Uni-A (*p* < 0.001), and in the Multi-V than in the Multi-A (*p* < 0.001). There was also a significant main effect of task type (F_(1,50)_ = 9.37, *p* < 0.01, η_p_^2^ = 0.16), indicating that response time was significantly shorter in the unimodal task type (*p* < 0.01). 

There was a significant two-way interaction between the target modality and group (F_(1,50)_ = 5.90, *p* < 0.05, η_p_^2^ = 0.11) and between the target modality and task type (F_(1,50)_ = 84.48, *p* < 0.001, η_p_^2^ = 0.63) (Table 2). There was also a significant three-way interaction between the target modality, task type, and group (F_(1,50)_ = 4.07, *p* < 0.05, η_p_^2^ = 0.08). The interaction between task type and group was not significant (*p* > 0.05). Post hoc analysis using Bonferroni correction revealed that both groups performed better in unimodal than multimodal tasks in visual modality (i.e., better performance in Uni-V than Multi-V, *p* < 0.001). The reverse trend, however, was found in auditory modality. That is, both age groups spent less time in Multi-A than Uni-A (*p* < 0.001), but significance was observed only in the findings for the older group (*p* < 0.001). 

With regards to performance accuracy, as shown in Figure 5b, we confirmed a similar trend as noted with response time. There was a significant main effect of group (F_(1,50)_ = 81.49, *p* < 0.001, η_p_^2^ = 0.62) and target modality (F_(1,50)_ = 162.99, *p* < 0.001, η_p_^2^ = 0.77). The findings indicated that younger adults performed significantly better (*p* < 0.001) and both groups performed better in the visual (i.e., Uni-V and Multi-V) than in the auditory modality (Uni-A and Multi-A, *p* < 0.001). Moreover, there was a significant two-way interaction effect between the target modality and group (F_(1,50)_ = 71.94, *p* < 0.001, η_p_^2^ = 0.59). Post hoc analysis revealed that the performance difference between groups was much larger in auditory (Uni-A and Multi-A, *p* < 0.001) than in visual modality (Uni-V and Multi-V, *p* < 0.05). Unlike the findings for response time, a three-way interaction was not significant (*p* > 0.05). 

Collectively, our behavioral data analysis indicated the dominance effect of visual modality and the effect of aging on cognitive function. Interestingly, we found a modality-specific effect on multisensory task performance only in the older adult group. That is, auditory target identification (Multi-A) was enhanced by visual non-target information, while visual target identification (Multi-V) was hampered by auditory non-target information only in the older adults. How this happens is further analyzed in the next section. 

### 3.2. Examining the Role of Unattended Stimuli in Multisensory Integration: Reference or Distraction? 

Our behavioral findings indicated that older adults exploited a modality-specific effect in multisensory information. To determine if unattended stimuli play a certain role of ‘reference’ or ‘distraction’, we analyzed the influence of visual information processing on enhancement between Uni-A and Multi-A, because no significant enhancement between Uni-V and Multi-V was found (see Figure 5a). Note that visual information was a distracting stimulus and auditory information was the target in the Multi-A condition. However, the older adult group seemed to exploit the visual information as a ‘negative’ reference rather than being distracted. 

For this assessment, we recoded a new variable by subtracting the response time of Multi-A from that of Uni-A (i.e., ΔUni-A–Multi-A). In our hypothesis, the Multi-A condition reveals the occurrence of auditory selective attention and visual inhibition at the same time. Therefore, those who performed better for the visual non-target stimuli against the auditory target stimuli would be better at visual inhibition. This indicates that these people have a special visual inhibition capability, so they can exploit this capability as a negative reference information to detect the auditory target. Conversely, those who do not have this special visual inhibition capability might suffer more from the distracting visual non-target information. 

In order to test this hypothesis, we grouped all the participants into three subgroups using the response time in the Multi-A condition: ‘high’ for those who scored in the highest quartile, ‘low’ for those who scored in the lowest quartile, and ‘middle’ for the rest of them. The grouping criterion followed the criterion frequently used in the education research [44]. 

In Table 3, within those who performed poorer in the Multi-A (i.e., ‘Low’), the older adults showed significant enhancements (i.e., 38.65 for younger adults and 1230.85 for older adults). Thus, even the older adults who performed poorly in the Multi-A showed a certain modality-specific effect, and the visual non-target might have not been causing deterioration. We performed independent sample *t*-tests between younger and older adult groups in each subgroup. The high and middle performance subgroups in the Multi-A were not significantly different between the younger and older adult groups; however, the older adults in the low performing subgroup showed significant enhancement with the visual non-target (*p* < 0.05). 

This finding may indicate that an ability to deal with distracting visual information in a multimodal context plays an important role in the Multi-A enhancement, specifically in the older adults group. This hints that though the older adults showed an obvious decline in general inhibitory control as compared to the younger adults, the loss of inhibition control can be partially compensated for by employing visual stimuli as a ‘negative’ reference. 

## 4. Discussion 

The present study examined the effect of ageing in multisensory information processing. The younger and older adult groups showed a shared but distinctive tendency of performance. First, both groups outperformed with visual contour identification presented in a unimodal context. Second, the auditory stimuli alone (i.e., Uni-A) were not sufficiently salient for identifying the contour direction. However, when visual non-target stimuli were given with auditory target identification, the response time was shortened. This trend was more obvious in the older than in the younger adult group, indicating that the older adults were more supported by the visual distracting information, which showed their special modality-specific multisensory information processing. Our findings suggest that the loss of inhibitory control with ageing can benefit from a special modality of distraction. This interpretation may be a result of the nature of our experimental apparatus (i.e., AV-CIT). The comparison between our experimental data with scores from a computerized version of Stroop Word Color Test [45] were not significantly different (see Table 4). 

### 4.1. Vision is Dominant, So Auditory Distraction Is Less Prominent in the Visual Target Identification

Vision is dominant, so auditory distraction is less prominent in visual target identification. Our findings again confirmed that both younger and older groups required significantly less time when the target was presented in the visual modality and this trend was the same between the younger and older adult groups. In effect, it seemed that visual stimuli play a leading role in object recognition [46,47,48,49], possibly because the greater specificity [50] of an icon or image can provide detailed information for identifying the object while a sound is relatively abstract. Diaconescu et al. suggested that our visual perception is dominant in comparison with other sensory modalities [25]. From an evolutionary perspective, this specificity is a necessity of the phylogenetic or developmental medium and, as a consequence, endows priority in processing and recognizing visual information [51]. Visual information is hard-wired to obtain primary access to our attentional resources and this tendency remains until late adulthood. The current findings were in line with Guerreiro and her colleague’s findings [7,31,32], in which the dominance of visual over auditory information remained until later adulthood, although it decreased severely with ageing. 

In addition, the finding that auditory distraction did not severely deteriorate visual selective attention was possibly due to the distinct filtering mechanisms within the visual and auditory modalities, which might be differentially affected by ageing. Specifically, auditory distractors appear to be filtered out at both central [52,53] and peripheral neurocognitive levels [19,54], predominantly during cross-modal visual selective attention [52]. In contrast, visual distraction appears to be filtered out only at the central (e.g., visual cortex) processing levels [55], with the largest attentional modulations occurring at the highest levels of the visual processing hierarchy [56]. Thus, with ageing, central inhibitory processing is more affected than the peripheral, and auditory distraction did not severely deteriorate visual selective attention. 

This account is also confirmed by Stothart and Kazanina [39]. They conducted an ERP study to examine peripheral and cortical involvement of auditory interference between age groups. Their findings showed that an increased vulnerability to auditory distraction was due to the cortical inability to inhibit peripheral processing. The combination of increased early sensory responses P50 and N1, absent or strongly reduced N2 to standard stimuli, and an increased P3a to deviant stimuli in older participants points to a decreased ability to inhibit responses to regular repeating information, and greater attentional capture from rare task-irrelevant information. In addition, Odegaard et al. [57] reported that visual dominance still existed but that the magnitude of the difference decreased in the multisensory condition, interpreting the enhancement observed in our study. 

### 4.2. Visual Distraction as a Negative Reference for Auditory Target Identification

Both age groups spent less time in Multi-A than Uni-A, while they spent more time in Multi-V than Uni-V. The findings indicated that auditory distraction seemed to hamper the participants’ performances, while visual distraction played a different role in that case. The current findings importantly suggested that when presented with visual distraction, auditory target detection was enhanced, while visual target detection was hindered by auditory distraction. This asymmetric interaction is inconsistent with the previous findings reporting that older adults were more susceptible to task-irrelevant auditory suppression during visual attention tasks than task-irrelevant visual suppression during auditory attention tasks [7,58]. 

One plausible explanation can be the role of visual stimuli as a negative reference. Note that in our study, the Multi-A condition used the auditory contour stimuli as a target and the visual contour as a non-target, by which the participants seemed to use visual information as a negative feedback reference item to quickly identify the target auditory contour direction. The more interesting finding is that this observation was noted in the older adult group. Amer et al. [59] also noted the same outcomes, that age-related susceptibility to distracting information can, counterintuitively, benefit older adults more than younger adults in cognitive performance. For instance, Biss et al. [60] examined the effect of context dependency on recall between younger and older adults using same or different lists of words as distractors. Their finding showed that the performance of older adults was enhanced when the same list of words was presented as a distractor, indicating that the cognitive decline in older adults (i.e., the increased distractibility to process both relevant and irrelevant stimuli equally) opened a chance to learn a previously presented list of words through distractors. Lee et al. [61] reported that implicit visual memory is enhanced when information is recognized and encoded in a holistic manner (i.e., the reduced attentional control). This is evidenced by decreased activation of the dorsolateral prefrontal cortex (DLPFC), which indicates the low cognitive load imposed to process the given task. In a similar vein, Lourenco and Maylor [62] examined the effect of presenting distracting information and identified the role of prospect memory benefits for older adults. The previous findings suggested that older adults fanned out their attentional focus over relevant auditory and irrelevant visual stimuli as well, while increasing the chance to benefit from the unnecessary information that might be relevant depending on the context [59]. 

In brain studies, the principle of inverse effectiveness well represents the fact that a decrease in sensory perception increases the magnitude of multisensory enhancements [63]. Neuro-imaging studies have reported that deactivations in the brain are associated with age-related cognitive declines and are prominent in the primary sensory cortices [3,5,20,21,22,23,24]. Interestingly, additional enhancement in several brain activation networks were found in the older adults [2,6,25], implying the development of a compensatory strategy to cancel out the neurological deactivation in sensory perception of the elderly. In addition, Stothart and Kazanina [39] reported N1 component enhancement (i.e., early selective processing) in the older adult group, while the younger age group did not seem to employ the same strategy. Moreover, a theory of “increased noise at baseline” demonstrated that older adults utilized their way of weighing information when information is unreliable or important [63]. In doing so, older adults were shown to recruit more multisensory brain areas than younger adults [64,65,66], which increased the brain activation, specifically in the frontal areas, from baseline [18]. In addition, functional magnetic response imaging (fMRI) evidence showed an alteration in the balance between a resting state and a task-involved activation. That is, increased default mode network (DMN) activity was observed in task-relevant areas (i.e., the dorsolateral prefrontal cortex) during task performance [67,68]. 

## 5. Conclusions

This study suggests that the younger and older adult groups showed a shared but distinctive tendency of performance in the multisensory information processing. Although performance of visual target identification was impeded by auditory distraction, performance of auditory target identification was enhanced by visual distraction. This trend was prominent in the older adult group, implying that the loss of visual inhibitory control supported the older adults’ modality-specific multisensory information processing. 

There are some limitations that limit the generalizability of the findings. First, we recruited younger and older adults whose mean ages were in the mid-20s and mid-50s, respectively. More confirmation with data from patients aged over 65 years is urgently needed. A relatively small sample size would be problematic, so a scaled-up experiment is indeed planned soon. A similar study could also be performed in a simulated real-world situation employing various modalities at the same time (i.e., multisensory integration in a virtual reality system). Another type of future study might be the use of AV-CIT in the patients with cognitive impairment (e.g., dementia, mild cognitive impairment). 

## Figures and Tables

**Table 1:** Descriptive statistics of response time and accuracy across presentation time.

		Presentation Time Mean (M)/Standard Deivation (SD)					
		500	1000	1500	2000	2500	3000
Response time (RT) (ms)	Younger	3719.20	3220.79	2929.75	2825.18	2580.51	2385.46
	(N = 30)	(1436.89)	(1278.81)	(1054.31)	(1093.25)	(916.28)	(865.08)
	Older	4262.778	3843.881	3536.023	3501.994	3145.986	2952.384
	(N = 22)	(3421.64)	(1702.78)	(1484.28)	(1695.64)	(1465.81)	(1461.03)
Accuracy (%)	Younger	84.83	94.33	95.83	96.00	96.50	97.50
	(N = 30)	(23.36)	(14.60)	(10.03)	(12.07)	(9.35)	(6.91)
	Older	62.05	68.18	70.91	73.18	75.91	75.45
	(N = 22)	(33.04)	(30.49)	(33.80)	(33.71)	(29.78)	(30.58)

**Table 2:** Descriptive statistics of response time and accuracy across audio-visual contour identification task.

		AV-CITs M(SD)			
		Uni-A	Uni-V	Multi-A	Multi-V
RT (msec)	Younger	3849.80	1752.69	3608.48	2595.58
	(N = 30)	(746.51)	(383.34)	(569.12)	(307.56)
	Older	4989.42	1993.77	4240.48	2938.36
	(N = 22)	(1829.16)	(464.93)	(1027.51)	(587.92)
Accuracy (%)	Younger	90.83	98.61	89.03	99.58
	(N = 30)	(10.51)	(4.68)	(12.21)	(1.27)
	Older	51.14	94.51	49.81	97.35
	(N = 22)	(22.24)	(7.43)	(21.69)	(3.97)

**Table 3:** Enhancement between Uni-A and Multi-A in the Multi-A condition.

Subgroups on Response Time in the Multi-A Condition	ΔUni-A–Multi-A			
	Younger (N = 30) M(SD)		Older (N = 22) M(SD)	
High	392.66	(183.91)	474.99	(311.43)
Middle	270.66	(506.92)	624.14	(660.79)
Low	38.65	(953.34)	1230.85	(2297.46)

N = 8 for high and low groups of younger adults, and N = 6 for high and low groups of older adults, respectively.

**Table 4:** Correlation between audio-visual contour identification task and Stroop Word Color Test.

Condition	Stroop Task	Uni-A	Uni-V	Multi-A	Multi-V
1	Color naming	0.30 *	0.34 *	0.24	0.26
2	Word reading	0.63 **	0.37 **	0.56 **	0.48 **
3	Word reading-Congruent	0.19	0.33 *	0.17	0.30 *
4	Word reading-Incongruent (Color inhibition)	0.46 **	0.36 **	0.56 **	0.21
5	Color reading-Incongruent (Word inhibition)	0.32 *	0.47 **	0.49 **	0.35 **

N = 52 for this correlation analysis. ** *p* < 0.01, * *p* < 0.05.
